# Ultrasensitive Label-Free Electrochemical Detection of *Pseudomonas aeruginosa* Using a Surface Molecularly Imprinted Polymer-Modified Screen-Printed Electrode

**DOI:** 10.3390/polym18121465

**Published:** 2026-06-11

**Authors:** Naphatsawan Vongmanee, Jindapa Nampeng, Chuchart Pintavirooj, Sarinporn Visitsattapongse

**Affiliations:** Department of Biomedical Engineering, School of Engineering, King Mongkut’s Institute of Technology Ladkrabang, Bangkok 10520, Thailand; naphatsawan.v@hotmail.com (N.V.); jindapa.na@kmitl.ac.th (J.N.); chuchart.pi@kmitl.ac.th (C.P.)

**Keywords:** *Pseudomonas aeruginosa*, electrochemical biomimetic sensor, molecularly imprinted polymer, screen-printed electrode, cyclic voltammetry, nosocomial infection, ultrasensitive detection

## Abstract

*Pseudomonas aeruginosa* is a major opportunistic pathogen frequently associated with nosocomial infections, such as pneumonia, urinary tract infections, and wound infections, particularly in immunocompromised or hospitalized patients. These infections are often difficult to treat due to the pathogen’s intrinsic antibiotic resistance and biofilm-forming ability. Therefore, rapid and selective detection of *P. aeruginosa* is essential for early diagnosis and effective infection control. In this study, a novel surface-imprinted MIP design uniquely combines methacrylamide (MAM), acrylamide (AAM), and vinylpyrrolidone (VP) monomers to generate recognition cavities that are complementary to the surface morphology and physicochemical properties of *Pseudomonas aeruginosa* cells. Unlike traditional MIP approaches, this surface imprinting strategy provides improved stability and reproducibility, without relying on biological recognition elements like antibodies or aptamers. This novel approach enabled us to achieve an ultralow LOD of 1 CFU/mL over a linear range of 1–10^4^ CFU/mL, demonstrating excellent analytical performance. In addition, the sensor exhibited good reproducibility with an RSD of 5–12%. The novelty of this work lies in the use of a surface-imprinted MIP strategy combined with a multi-monomer system to enhance bacterial recognition and sensing performance. Overall, the proposed MIP-based electrochemical biomimetic sensor offers a rapid, cost-effective, and portable platform with strong potential for the detection of *P. aeruginosa* in clinical and environmental applications.

## 1. Introduction

*Pseudomonas aeruginosa (P. aeruginosa)* is an opportunistic Gram-negative pathogen that poses a serious threat to public health, particularly in immunocompromised patients [1,2,3]. It is a major cause of hospital-acquired infections [4], including pneumonia [5], urinary tract infections [6], sepsis [7], and chronic wound infections [8]. The intrinsic resistance of *P. aeruginosa* to multiple antibiotics, along with its ability to form biofilms, significantly complicates treatment and contributes to high morbidity and mortality rates [9,10,11]. Moreover, its pathogenicity is driven by various virulence factors, including biofilm formation, the secretion of exotoxins and the production of tissue-degrading enzymes such as elastase and proteases. These factors disrupt host cellular functions, damage tissue integrity, and trigger severe inflammatory responses, leading to persistent and difficult-to-treat infections. Therefore, the rapid and accurate detection of *P. aeruginosa* is crucial for effective clinical diagnosis and infection control.

Traditional detection methods, including bacterial culture techniques, polymerase chain reaction (PCR), and immunological assays, are widely applied for *P. aeruginosa* identification [12,13,14]. However, these techniques are often time-consuming, labor-intensive, and require sophisticated instrumentation and skilled personnel [15,16,17]. As a result, there is a growing interest in developing electrochemical biosensors as alternative diagnostic platforms due to their simplicity, low cost, high sensitivity, and compatibility with miniaturized and point-of-care devices [18,19,20,21].

Electrochemical biomimetic sensors have been widely explored for bacterial detection by converting biological interactions at the electrode surface into measurable electrical signals [22,23,24]. Among various electrochemical techniques, cyclic voltammetry (CV) is a powerful and widely used method for probing interfacial electron-transfer processes [25,26]. CV enables direct observation of changes in redox current that occur as a result of surface modification or target binding events, making it particularly suitable for monitoring bacteria sensor interactions in real time [27,28]. In this context, electrochemical biosensors have emerged as promising alternatives due to their high sensitivity, low cost, ease of miniaturization, and compatibility with point-of-care diagnostic systems [29,30].

Biomimetic sensors have emerged as promising tools for bacterial detection due to their rapid response, high sensitivity, and potential for real-time analysis [31,32,33]. Among various biorecognition elements, molecularly imprinted polymers (MIPs) have gained significant attention as artificial recognition elements in electrochemical biomimetic sensors [34,35]. MIPs are synthesized by polymerizing functional monomers and crosslinkers in the presence of a target template [36], followed by template removal to generate specific recognition cavities complementary to the target in terms of size, shape, and chemical functionality [37]. Compared with natural bioreceptors, MIPs exhibit superior chemical stability, mechanical robustness, and resistance to environmental variations, making them attractive for bacterial sensing applications [38,39].

Recently, molecularly imprinted polymer (MIP)-based biomimetic sensors have attracted significant attention for bacterial detection due to their high selectivity, chemical stability, and suitability for operation in complex matrices. In particular, MIPs offer a promising alternative to biological recognition elements, overcoming limitations such as poor stability, high cost, and limited shelf life associated with antibodies and aptamers. Recent advances have demonstrated that the integration of MIPs with electrochemical, optical, and mass-sensitive transducers enables rapid and sensitive detection of pathogenic bacteria, including *Pseudomonas aeruginosa*. Complementary approaches using advanced nanomaterials have further expanded biosensor capabilities such as machine learning-enabled smart non-invasive glucose monitoring systems using RF sensors [40], reduced graphene oxide-based absorbance biosensors for detecting *Escherichia coli* DNA [41], MXene-based platforms for biomedical and environmental applications [42], and functional black phosphorus-based sensors for food safety applications [43]. Despite these advances, challenges remain in achieving the highly selective, label-free, and reproducible detection of *P. aeruginosa*, particularly when targeting whole bacterial cells. The performance of MIP-based sensors is strongly dependent on the selection of appropriate functional monomers, which govern the formation of specific binding sites and overall recognition efficiency.

In this study, methacrylamide (MAM), acrylamide (AAM), and N-vinylpyrrolidone (VP) were selected as functional monomers due to their ability to form hydrogen bonding and polar interactions with biomolecular components on the surface of *P. aeruginosa* [44]. The combination of amide- and lactam-containing monomers is expected to enhance imprinting efficiency and improve selective recognition toward bacterial surface structures [45]. In this work, imprinting efficiency is defined as the effectiveness of the polymer in generating functional binding sites within the polymer matrix, which is evaluated by comparing the electrochemical response of the MIP with that of the corresponding non-imprinted polymer (NIP) under identical conditions. Selective recognition refers to the ability of the MIP to preferentially interact with *P. aeruginosa* over non-target bacteria, which is assessed by comparing the electrochemical responses (percent change in current) toward the target and non-target bacteria. Furthermore, a surface-imprinting strategy was employed to generate accessible recognition cavities, which is particularly advantageous for large targets such as bacterial cells. When integrated onto a screen-printed electrode (SPE), the MIP layer acts as a selective recognition interface. Upon binding of *P. aeruginosa* within the imprinted cavities, electron transfer between the electrode surface and the redox probe is hindered, resulting in a measurable decrease in peak current in cyclic voltammetry (CV). This label-free detection mechanism enables quantitative analysis without the need for additional reagents or complex signal amplification steps.

In this work, we report a surface-imprinted MIP-modified electrochemical biomimetic sensor for the detection of *P. aeruginosa* using cyclic voltammetry as the signal transduction technique. Compared with previously reported systems, the proposed approach offers improved stability, cost-effectiveness, and simplified fabrication while maintaining high sensitivity and selectivity. The sensor’s electrochemical behavior, analytical performance, and selectivity toward *P. aeruginosa* are systematically evaluated, demonstrating its potential for rapid detection.

## 2. Materials and Methods

### 2.1. Bacteria Preparation

*P. aeruginosa* was used as the target bacterial species in this study. The bacterial strain was obtained from a certified microbial culture collection (ATCC 27853, Manassas, VA, USA). A single colony was selected and inoculated into 10 mL of sterile LB broth (L3522, Merck, Darmstadt, Germany), followed by incubation at 37 °C with shaking at 240 rpm for 15 h to obtain an overnight culture [44]. The bacterial suspension was subsequently harvested by centrifugation at 4000 rpm for 5 min and washed three times with sterile phosphate-buffered saline (PBS, pH 7.4) (P4417, Merck, Darmstadt, Germany) to remove residual culture medium components. The washed bacterial pellet was resuspended in PBS. The concentration of bacterial suspensions was estimated by measuring the optical density at 600 nm (OD_600_) using a UV–Vis spectrophotometer. Bacterial cultures were grown to the exponential phase, collected, and diluted with sterile phosphate-buffered saline (PBS) to obtain the desired OD values [44,45]. The initial bacterial concentration was calculated using a previously established OD_600_ to colony-forming unit (CFU) calibration curve, where OD_600_ = 1.0 corresponds to approximately 1.56 × 10^8^ CFU/mL.

**Figure 1:**
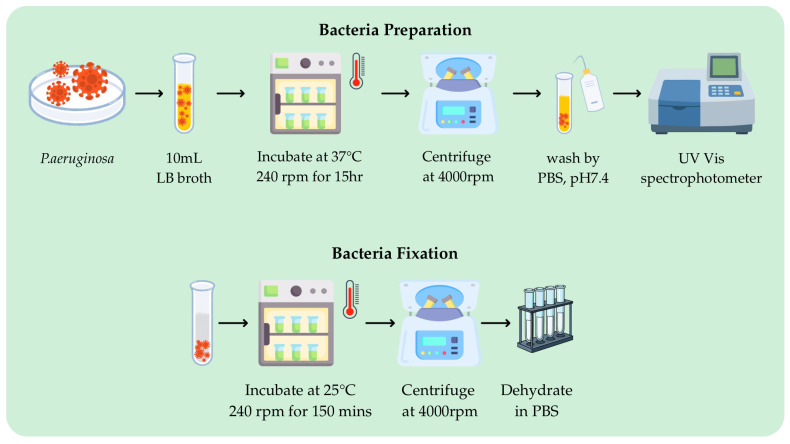
Schematic illustration of bacterial preparation protocol.

For bacterial fixation, the washed bacterial pellet was resuspended in 2.5% glutaraldehyde (354400, Sigma-Aldrich, St. Louis, MO, USA) solution and incubated at room temperature for 150 min to inactivate the bacteria and preserve their morphology. After fixation, the bacterial cells were collected by centrifugation at 4000 rpm for 5 min and washed three times with sterile phosphate-buffered saline (PBS) to remove residual fixative. The fixed bacteria were subsequently dehydrated through a graded ethanol series of 50%, 60%, 70%, 80%, and 90% for 5 min at each concentration, followed by two dehydration steps in 100% ethanol for 15 min each (459836, Sigma-Aldrich, St. Louis, MO, USA). Finally, the dehydrated bacterial cells were resuspended in PBS and used for subsequent experiments. The fixation of *P. aeruginosa* cells using glutaraldehyde, followed by ethanol dehydration, was performed to preserve cell morphology and surface structure during the imprinting process, ensuring the formation of well-defined recognition sites in the MIP layer as shown in Figure 1. Serial dilutions were then prepared in PBS to obtain bacterial suspensions with concentrations ranging from 1 to 10,000 CFU/mL for electrochemical measurements. All bacterial suspensions were freshly prepared prior to use to ensure sample consistency. All sensing experiments were performed under identical experimental conditions to ensure reproducibility.

### 2.2. Polymer Synthesis for Detection

The polymer used for bacterial detection was synthesized by free-radical polymerization using methacrylamide (MAM) (109606 Sigma-Aldrich, St. Louis, MO, USA), acrylamide (AAM) (A8887, Sigma-Aldrich, St. Louis, MO, USA), and N-vinylpyrrolidone (VP) (434450, Merck, Darmstadt, Germany) as functional monomers. To investigate the effect of polymer composition on sensing performance, five different polymer synthesis conditions were prepared by varying the feed ratios of MAM, AAM, and VP, as summarized in Table 1.

For each synthesis condition, the monomers were mixed with 45 mg of *N*,*N*′-(1,2-dihydroxyethylene)bisacrylamide (DHEBA) (294381, Sigma-Aldrich, St. Louis, MO, USA) as a crosslinker and 1.5 mg of azobisisobutyronitrile (AIBN) (768375, Sigma-Aldrich, St. Louis, MO, USA) as a radical initiator. All chemical substances were dissolved in 300 μL of dimethyl sulfoxide (DMSO) (W387509, Sigma-Aldrich, St. Louis, MO, USA) to obtain a homogeneous solution. The polymerization reaction was then carried out under controlled temperature conditions at 80 °C for 5 min to obtain the pre-polymer solution. After polymerization, the pre-polymer solution was allowed to cool to room temperature or placed in an ice bath. The resulting pre-polymer solutions from each synthesis condition were collected and stored at 4 °C prior to being imprinted onto the working electrode.

### 2.3. Screen Printed Electrode (SPE) Preparation

The screen-printed electrode (SPE; DRP-220BT) used in this study consisted of gold working and counter electrodes and a silver reference electrode. The working electrode had a diameter of 4 mm and was used as the active area for polymer modification.

A defined volume 1 µL of the pre-polymer solution from Section 2.2 was drop-cast onto the surface of the working electrode and allowed to partially dry at room temperature. Subsequently, a 1 µL aliquot of the *P. aeruginosa* template solution was drop-cast onto the pre-polymer layer on the working electrode. The electrode was then exposed to UV irradiation (365 nm) for 3 h to initiate polymer crosslinking and imprinting in the presence of the bacterial template.

**Figure 2:**
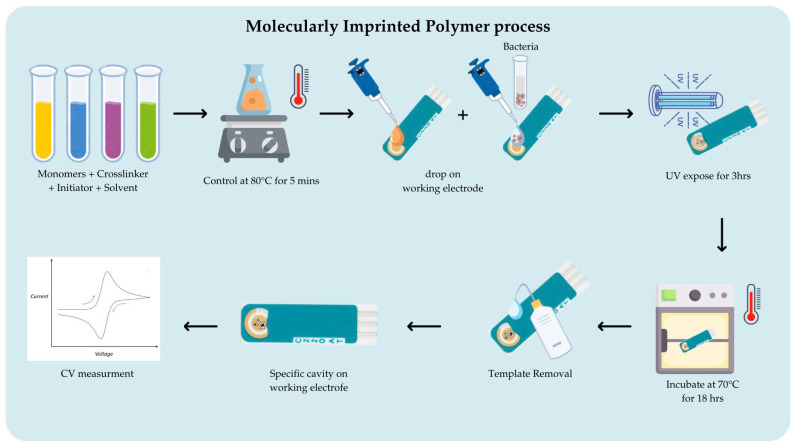
Schematic illustration of the molecularly imprinted polymer (MIP) fabrication process. Arrows indicate the sequence of the experimental steps.

After UV exposure, the modified electrodes were further cured in a heated oven at 70 °C for 18 h to complete polymerization and enhance polymer stability. The resulting polymer-imprinted SPEs were allowed to cool to room temperature and subsequently subjected to template removal. The bacterial template was removed by washing the electrodes with 10% (*v*/*v*) acetic acid for 30 min, followed by soaking in Milli-Q (MQ) water for 30 min [45]. The SPE were then allowed to dry completely at room temperature prior to electrochemical measurements as shown the procedure in Figure 2.

### 2.4. Cyclic Voltammogram Measurement

Cyclic voltammetry (CV) measurements were performed to evaluate the electrochemical behavior of the polymer-modified screen-printed electrodes (SPEs) before and after *P. aeruginosa* detection. All electrochemical experiments were carried out using a potentiostat (μStat-i 400, Metrohm Dropsens, Asturias, Spain) controlled by DropView 8400 software (Metrohm Dropsens, Asturias, Spain). CV measurements were conducted in a redox probe solution containing the [Fe(CN)_6_]^3−^/[Fe(CN)_6_]^4−^ as a redox couple solution. The potential was swept over the range of −0.3 to +0.6 V at a fixed scan rate of 50 mV s^−1^. For sensing measurements, the bacterial suspension was directly dropped onto the SPE surface, and the electrochemical response was recorded immediately without a separate incubation step. The same SPE was used for measurements across different concentrations, and the electrode was rinsed with deionized water between each measurement to minimize potential carryover effects. The CV responses were recorded to monitor changes in peak current associated with bacterial rebinding on the working electrode surface. Electrochemical measurements were performed immediately after sample deposition onto the electrode surface without additional incubation to evaluate the rapid detection capability of the sensing platform. The electrochemical signals obtained from the polymer-imprinted SPEs were quantitatively compared with those of non-imprinted and bare SPEs based on the percent change in peak current. This comparison was used to evaluate the sensing performance and to assess selectivity by distinguishing the response toward *P. aeruginosa* from that of non-target bacteria.

## 3. Results

### 3.1. Electrochemical Detection of P. aeruginosa Using Polymer-Imprinted SPE

The electrochemical responses of polymer-imprinted SPEs synthesized under five different polymerization conditions were compared using cyclic voltammetry (CV). Figure 1 illustrates the CV curves obtained from each polymer synthesis condition after rebinding with *P. aeruginosa*. Distinct differences in peak current changes were observed among the five conditions, indicating that polymer composition plays an important role in the sensor response toward the target bacteria. Among the tested conditions, certain polymer formulations exhibited a more pronounced decrease in peak currents upon bacterial rebinding, indicating a higher electrochemical response toward the target bacteria. In contrast, other conditions showed relatively smaller current changes, suggesting a comparatively lower response under the same conditions. These variations may be attributed to differences in the effectiveness of imprinting and the accessibility of recognition sites, although direct binding interactions were not measured in this study. The percent change in peak current was calculated to quantitatively compare the sensor response of each polymer synthesis condition, as summarized in Table 2.

**Figure 3:**
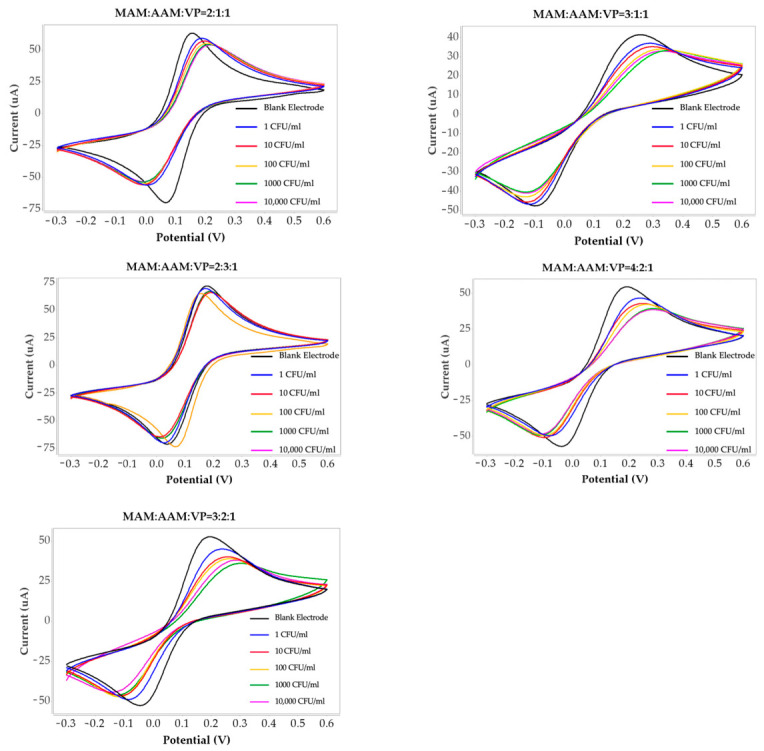
Cyclic voltammograms obtained for five polymer synthesis conditions recorded in the potential range from −0.3 to 0.6 V at a scan rate of 50 mV s^−1^. Condition 1: MAM:AAM:VP = 2:1:1, Condition 2: MAM:AAM:VP = 3:1:1, Condition 3: MAM:AAM:VP = 2:3:1, Condition4: MAM:AAM:VP = 4:2:1 and Condition 5: MAM:AAM:VP = 3:2:1. Different current scales were applied for each panel to enhance visualization of signal changes.

The electrochemical responses of polymer-imprinted on SPEs synthesized under five different polymerization conditions were compared using cyclic voltammetry (CV). Figure 3 illustrates the CV curves obtained from each polymer synthesis condition after rebinding with *P. aeruginosa*. Distinct differences in peak current changes were observed among the five conditions, indicating that polymer composition played a critical role in bacterial recognition and sensing performance. Among the tested conditions, certain polymer formulations exhibited a more pronounced decrease in peak currents upon bacterial rebinding, indicating a higher electrochemical response toward the target bacteria. In contrast, other conditions showed relatively smaller current changes, suggesting a comparatively lower response under the same conditions These variations may be attributed to differences in the effectiveness of imprinting and the accessibility of recognition sites, although direct binding interactions were not measured in this study. The percent change in peak current was calculated to quantitatively compare the sensing performance of each polymer synthesis condition as summarized in Table 2.

**Figure 4:**
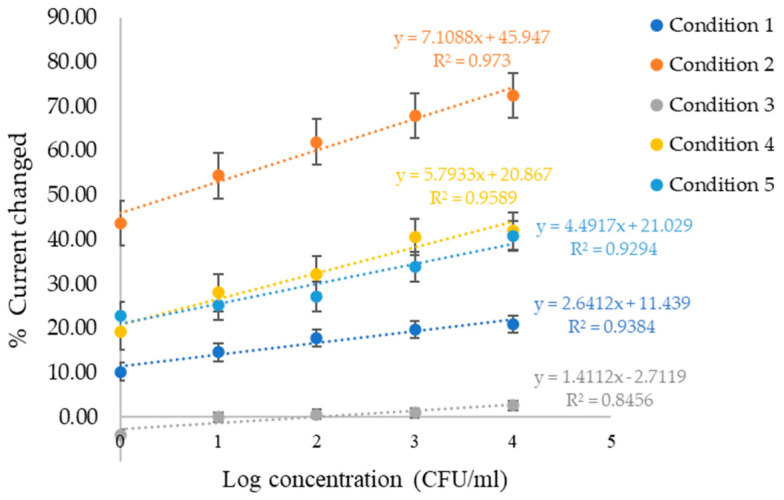
Calibration linearity ranges obtained from polymer-imprinted SPE prepared under different synthesis conditions.

In addition, the linearity ranges obtained from the five polymer synthesis conditions are compared in Figure 4. A linear relationship between the percentage current change and the logarithm of bacterial concentration was observed over the tested concentration range. This semi-logarithmic behavior may be associated with heterogeneous surface interactions and progressive occupation of the imprinted binding sites at increasing bacterial concentrations. Among the tested conditions, Condition 2 demonstrated the highest sensitivity with a slope of 7.1088 and a correlation coefficient (R^2^) of 0.9730, indicating superior electrochemical response toward *P. aeruginosa*. This enhanced performance reflects an optimal balance between polymer permeability and binding site accessibility, which facilitated efficient bacterial recognition and effective modulation of electron transfer. Based on these results, Condition 2 was selected as the optimal polymer formulation for subsequent sensing and selectivity studies. These findings clearly demonstrate that variations in monomer composition significantly influence the electrochemical behavior of the polymer-imprinted SPEs and highlight the importance of polymer synthesis optimization for achieving enhanced detection performance.

### 3.2. Selective Detection of P. aeruginosa Using Polymer-Imprinted SPE

The selectivity of the polymer-imprinted on SPEs toward *P. aeruginosa* was evaluated by comparing their electrochemical responses with those of selected non-target bacterial species and non-imprinted polymer (NIP) measurement. *Klebsiella pneumoniae* and *Haemophilus influenzae* were selected as non-target bacteria because they are Gram-negative pathogens with comparable bacterial size and morphology and are of clinical relevance, particularly in respiratory tract infections where co-infection with *P. aeruginosa* may occur in clinical samples.

**Figure 5:**
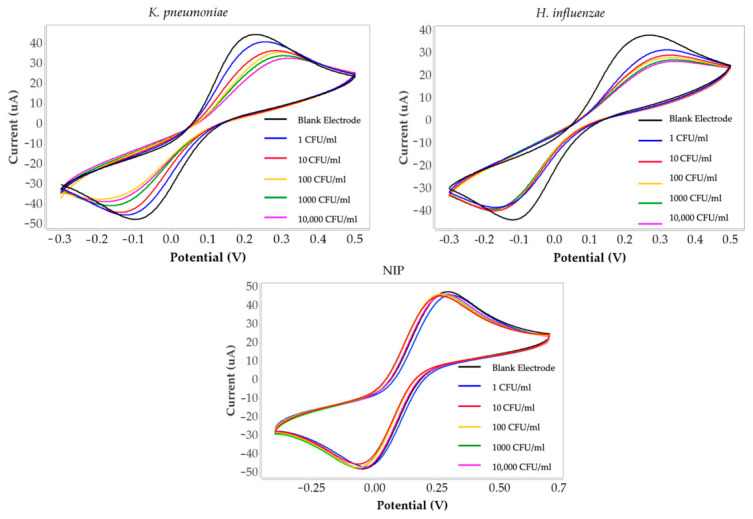
Cyclic voltammograms recorded using polymer-imprinted SPE prepared under Condition 2 in the potential range from −0.3 to 0.5 V at a scan rate of 50 mV s^−1^ for the detection of non-target bacteria (*K. pneumoniae* and *H. influenzae*) and NIP.

Figure 5 presents the CV responses obtained from measurements with non-target bacteria and NIP detection, while Table 3 summarizes the peak current values and percent changes in peak current recorded at different concentration levels after exposure to non-target bacteria and NIP under identical experimental conditions. The polymer-imprinted SPEs exhibited a significantly larger decrease in peak current in the presence of *P. aeruginosa* compared with *K. pneumoniae*, *H. influenzae* and NIP, indicating preferential binding of the target bacteria to the imprinted cavities.

**Figure 6:**
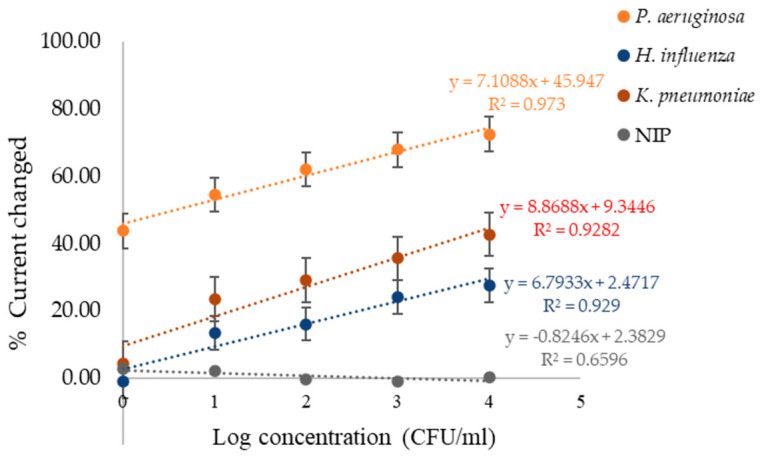
Comparison of calibration linearity ranges for target and non-target bacteria and NIP measurement. Error bars represent the standard deviation of triplicate measurements (*n* = 3).

From Table 3, calibration curves were constructed by plotting the log concentration level of bacteria versus the percent change in current. As shown in Figure 6, the calibration curve for *P. aeruginosa* is consistently positioned above those of *K. pneumoniae* and *H. influenzae* across the investigated concentration range from 1 to 10,000 CFU/mL, indicating a higher overall electrochemical response resulting from specific binding to the imprinted cavities. In contrast, exposure to the non-target bacteria resulted in considerably smaller current changes, suggesting limited nonspecific adsorption on the polymer surface. The optimized polymer in Condition 2 demonstrated the highest selectivity, with a markedly higher percent change in current changed toward *P. aeruginosa* than toward *K. pneumoniae* and *H. influenzae*.

Although the calibration slope obtained for *K. pneumoniae* is slightly higher than that of *P. aeruginosa*, at 8.8688 and 7.1088, respectively, the absolute signal changes for *K. pneumoniae* remain significantly lower across the entire concentration range. This indicates that the interaction between the MIP layer and *P. aeruginosa* is stronger and more specific. The higher slope observed for *K. pneumoniae* may be attributed to nonspecific adsorption effects, which can induce relatively rapid signal changes with increasing concentration but do not result in stable or specific binding interactions. Therefore, the higher response magnitude and clear signal separation observed for *P. aeruginosa* confirm the selective recognition capability of the MIP-modified SPE.

In comparison, the NIP exhibited significantly lower current responses across all tested concentrations, with no distinct discrimination between target and non-target bacteria. This behavior confirms that the signal enhancement observed in the MIP-modified electrode arises from the presence of specific imprinted binding sites rather than the intrinsic properties of the polymer matrix. Therefore, the higher response magnitude and clear signal separation observed for *P. aeruginosa*, together with the minimal response of the NIP, confirm the selective recognition capability of the MIP-modified SPE.

These findings can be attributed to the specific recognition sites formed during the imprinting process, which provide complementary size, shape, and functional group interactions with *P. aeruginosa*. Overall, the results confirm that the polymer-imprinted SPEs enable selective electrochemical detection of *P. aeruginosa*, even in the presence of clinically relevant non-target bacteria.

### 3.3. Image Characterization for P. aeruginosa Detection

**Figure 7:**
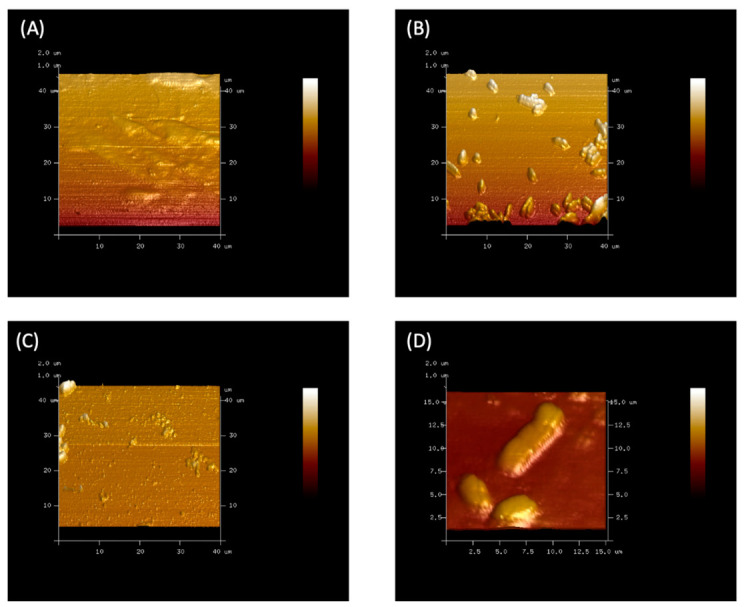
AFM images of the polymer-imprinted SPE at different stages: (**A**) polymer-coated SPE surface, (**B**) after *P. aeruginosa* template imprinting, (**C**) after template removal, and (**D**) after rebinding with P. aeruginosa. The disappearance and reappearance of bacterial-like surface features indicate successful template removal and selective rebinding of the target bacteria.

Image characterization was performed using atomic force microscopy (AFM) to visually confirm the imprinting process and the selective rebinding of *P. aeruginosa* on the polymer-imprinted SPE surface. The surface morphologies of the polymer-imprinted SPE and bacteria template imprinted surface after template removal and after rebinding of *P. aeruginosa* were systematically examined as shown in Figure 7.

AFM images illustrating the surface morphology changes in the polymer-imprinted SPE during different stages of the imprinting and rebinding process. As shown in Figure 7A, the polymer-coated SPE exhibited a relatively smooth and uniform surface, indicating homogeneous polymer coverage on the electrode. After bacterial template imprinting, shown in Figure 7B, distinct protrusions with sizes comparable to *P. aeruginosa* cells were observed on the polymer surface, confirming the successful immobilization of bacterial templates within the polymer matrix. Following the template removal process shown in Figure 7C, these protrusions were markedly reduced or absent, suggesting effective removal of surface-bound bacteria. Although well-defined cavity structures were not clearly visualized, the disappearance of these bacterial-like features suggests the formation of imprinted sites within the polymer layer, which may be attributed to the resolution limitations of AFM and the soft nature of the polymer matrix. Upon rebinding with *P. aeruginosa*, as shown in Figure 7D, bacterial-like structures reappeared on the polymer surface, indicating the ability of the polymer-imprinted SPE to recapture the target bacteria. This observation is consistent with the electrochemical results and supports the successful imprinting and rebinding behavior of the sensing platform. Although quantitative roughness parameters were not available, the observed changes in surface morphology qualitatively support the formation and rebinding characteristics of the imprinted polymer.

### 3.4. Limit of Detection (LOD) Calculation

Based on the calibration curve obtained under polymer synthesis Condition 2, the limit of detection (LOD) for *P. aeruginosa* was determined to be 1.00 CFU/mL (S/N = 3), demonstrating the high sensitivity of the polymer-imprinted SPE platform. The LOD was calculated based on the signal-to-noise ratio (S/N = 3). The standard deviation of the blank signal (σ) was obtained from three independent measurements using the polymer-imprinted SPE in the absence of bacteria. The blank measurements were performed in the redox probe solution without bacterial cells. The LOD was calculated using the equation LOD = 3σ/slope, where the slope was derived from the linear regression of the calibration curve between the logarithm of bacterial concentration and the percent change in peak current.

### 3.5. Reproducibility of the Sensor Performance

**Figure 8:**
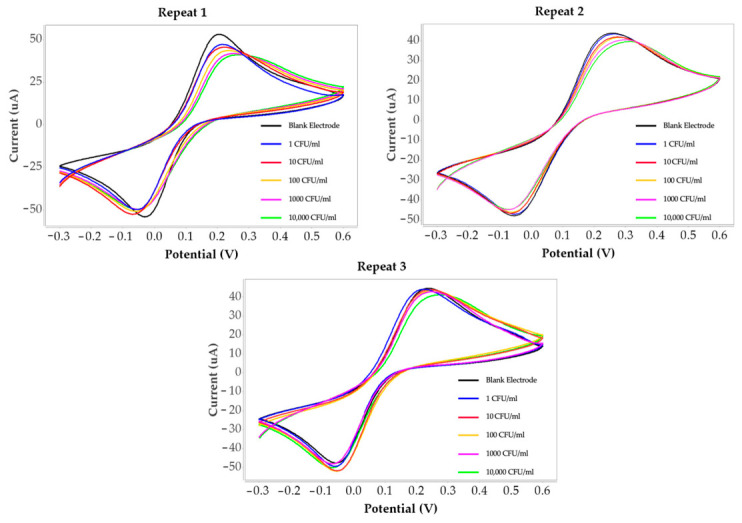
Cyclic voltammetry (CV) responses demonstrating the reproducibility of the sensor.

The reproducibility of the proposed sensing platform was evaluated using three independently prepared screen-printed electrodes (SPEs) under identical experimental conditions. All measurements were conducted following the same electrode modification protocol with same polymer in Condition 2. The electrochemical responses toward *P. aeruginosa* were examined over a range of concentrations, as shown in Figure 8, and Table 4 shows that average current response and consistent trends were observed across the three electrodes. The relative standard deviation (RSD) values were found to be within 5–12%, indicating good reproducibility of the MIP-modified SPEs. These results demonstrate reliable electrode-to-electrode performance and confirm the stability of the sensing platform.

The reproducibility of the MIP-based electrochemical biosensor was evaluated by calculating the relative standard deviation (*%RSD*). For each concentration of *P. aeruginosa*, three independent measurements were performed using separate electrodes. The *RSD* for each concentration was calculated as:

$$RSD\left(\%\right)=\frac{Standard\;deviation\;(SD)}{Average\;cueent\;response}\;\times \;100$$

The average *RSD* across all concentrations was then calculated to represent the overall reproducibility of the sensor. This approach ensures that the sensor’s performance is consistently assessed across the entire range of tested concentrations.

### 3.6. Comparison of Detection Performance for P. aeruginosa

As summarized in Table 5, the analytical performance of the proposed MIP-based electrochemical sensor was compared with previously reported methods for the detection of *P. aeruginosa.* The developed sensor achieved a limit of detection (LOD) of 1 CFU/mL over a linear range of 1–10^4^ CFU/mL, demonstrating competitive sensitivity among current electrochemical platforms. In comparison, lectin-based electrochemical biosensors and fluorescence-based methods typically exhibit higher detection limits of approximately 10 CFU/mL and 2 CFU/mL, respectively, with a dynamic range of 10–10^6^ CFU/mL [46,47]. In addition, conventional electrochemical sensors reported in the literature generally show detection limits in the range of 8–18 CFU/mL [48]. These results indicate that the proposed sensor provides a lower or comparable detection limit relative to several existing methods, highlighting its potential for sensitive bacterial detection.

## 4. Discussion and Conclusions

The selective detection of *P. aeruginosa* using the polymer-imprinted SPE is attributed to the formation of recognition cavities during the imprinting process, which are complementary in size, morphology, and surface chemistry to the target bacteria. Optimization of the monomer composition enabled the formation of binding sites that promote preferential interaction with *P. aeruginosa*, as reflecsted by a pronounced decrease in peak current during electrochemical measurements. Calibration analysis revealed that *P. aeruginosa* consistently generated a higher overall electrochemical response than non-target bacteria across the tested concentration range. This enhanced signal is attributed to efficient rebinding within the imprinted cavities which obstructs electron transfer between the redox probe and the electrode surface. This mechanism is consistent with MIP-based electrochemical sensing, where target binding induces steric hindrance and increases charge-transfer resistance.

In contrast, *K. pneumoniae* and *H. influenzae* showed significantly lower signal changes, indicating weaker and predominantly nonspecific interactions with the polymer layer. This observation is further supported by the response obtained from the corresponding non-imprinted polymer (NIP), which exhibited minimal current changes across all tested concentrations and showed no distinguishable selectivity toward *P. aeruginosa* over non-target bacteria. The absence of differential responses in the NIP confirms that nonspecific adsorption alone does not contribute significantly to the observed signal change. Therefore, the enhanced electrochemical response observed in the MIP-modified electrode can be directly attributed to the presence of specific imprinted cavities that facilitate selective rebinding of the target bacteria. The proposed sensing mechanism was interpreted based on the concentration-dependent electrochemical responses obtained from CV measurements. Although additional electrochemical characterization such as electrochemical impedance spectroscopy (EIS) could provide further insight into interfacial charge transfer processes, such measurements were not available in the present study and were therefore not performed. Nevertheless, the mechanistic interpretation is supported by the reproducible CV trends and is consistent with previously reported electrochemical biosensing systems.

The selectivity is strongly governed by the polymer composition, particularly the roles of MAM, AAM, and VP. Each monomer contributes to distinct physicochemical properties, including rigidity, hydrophilicity, and affinity toward bacterial surfaces. The optimized ratio (MAM:AAM:VP = 3:1:1) provides a balance between mechanical stability, cavity fidelity, and surface accessibility, thereby enhancing selective binding of *P. aeruginosa.* This balance accounts for the higher response magnitude observed for the target organism compared with non-target species, despite differences in calibration slopes.

The specificity of the recognition cavities arises from their structural and chemical complementarity to the surface of *P. aeruginosa*. Similarly to biological recognition systems such as antigen–antibody interactions, the imprinted cavities enable selective binding through a combination of shape matching and non-covalent interactions, including hydrogen bonding, electrostatic interactions, and hydrophobic effects.

A polymer-imprinted SPE platform was successfully developed for the selective electrochemical detection of *P. aeruginosa*. The polymer layer was synthesized via free-radical polymerization using MAM, AAM, and VP at a ratio of 3:1:1, and its performance was evaluated under five fabrication conditions. The optimized formulation yielded the highest overall signal response and the most distinct discrimination, indicating an optimal balance between rigidity, permeability, and binding site accessibility. The sensor demonstrated clear selectivity toward *P. aeruginosa* over non-target bacteria, including *K. pneumoniae* and *H. influenzae*. Although non-target species produced measurable responses, their absolute signal changes remained significantly lower. This highlights the importance of considering response magnitude and signal separation, rather than slope alone, when evaluating selectivity. The limit of detection was determined to be 1.00 CFU mL^−1^ (S/N = 3), demonstrating high sensitivity.

Compared with conventional methods, the proposed platform offers several advantages. Culture-based techniques require extended incubation times (24–72 h), limiting their applicability for rapid diagnostics, while molecular methods such as PCR, although sensitive, involve complex procedures, costly instrumentation, and trained personnel. In contrast, the polymer-imprinted SPE enables rapid, label-free detection through a simple electrochemical readout, reducing both analysis time and operational complexity. The use of synthetic recognition cavities eliminates the need for biological receptors, improving stability, reproducibility, and shelf life while lowering cost. Overall, the developed platform provides a simple, selective, and cost-effective approach for bacterial detection, with strong potential for point-of-care diagnostics and environmental monitoring.

## Figures and Tables

**Table 1:** Polymer synthesis conditions with different monomer ratios.

Condition	Molar Ratio (MAM:AAM:VP)	MAM (µL)	AAM (mg)	VP (µL)
1	2:1:1	10.7	14.2	10.7
2	3:1:1	21.4	7.1	10.7
3	2:3:1	21.4	21.3	10.7
4	4:2:1	32.1	14.2	10.7
5	3:2:1	10.7	7.1	10.7

**Table 2:** The percent changed in peak current for different polymer synthesis conditions.

Condition 1	Current (µA)	∆I (µA)	% Current changed
Blank	68.47		
1 CFU/mL	61.42	7.05	10.30
10 CFU/mL	58.46	10.01	14.62
100 CFU/mL	56.18	12.29	17.95
1000 CFU/mL	54.88	13.59	19.84
10,000 CFU/mL	54.17	14.31	20.89
Condition 2	Current (µA)	∆I (µA)	% Current changed
Blank	69.77		
1 CFU/mL	39.24	30.53	43.76
10 CFU/mL	31.76	38.02	54.49
100 CFU/mL	26.48	43.30	62.06
1000 CFU/mL	22.38	47.39	67.93
10,000 CFU/mL	19.13	50.64	72.59
Condition 3	Current (µA)	∆I (µA)	% Current changed
Blank	75.10		
1 CFU/mL	72.92	−2.90	−3.86
10 CFU/mL	70.02	0.00	0.01
100 CFU/mL	69.51	0.51	0.68
1000 CFU/mL	69.23	0.79	1.05
10,000 CFU/mL	68.01	2.01	2.68
Condition 4	Current (µA)	∆I (µA)	% Current changed
Blank	56.70		
1 CFU/mL	45.75	10.95	19.32
10 CFU/mL	40.76	15.94	28.11
100 CFU/mL	38.46	18.24	32.17
1000 CFU/mL	33.66	23.04	40.64
10,000 CFU/mL	32.87	23.83	42.02
Condition 5	Current (µA)	∆I (µA)	% Current changed
Blank	44.66		
1 CFU/mL	37.47	10.19	22.82
10 CFU/mL	36.40	11.26	25.21
100 CFU/mL	35.52	12.14	27.18
1000 CFU/mL	32.51	15.15	33.93
10,000 CFU/mL	29.39	18.27	40.92

**Table 3:** The percent changed in peak current of non-target bacteria and NIP detection.

*H. influenza*	Current (µA)	∆I (µA)	% Current changed
Blank	19.93		
1 CFU/mL	20.13	−0.20	−0.99
10 CFU/mL	17.25	2.68	13.45
100 CFU/mL	16.73	3.20	16.05
1000 CFU/mL	15.12	4.82	24.16
10,000 CFU/mL	14.43	5.51	27.62
*K. pneumoniae*	Current (µA)	∆I (µA)	% Current changed
Blank	35.14		
1 CFU/mL	33.58	1.56	4.43
10 CFU/mL	26.89	8.26	23.49
100 CFU/mL	24.89	10.25	29.17
1000 CFU/mL	22.64	12.50	35.57
10,000 CFU/mL	20.12	15.02	42.74
NIP	Current (µA)	∆I (µA)	% Current changed
Blank	42.31		
1 CFU/mL	41.12	1.20	2.82
10 CFU/mL	41.47	0.84	1.99
100 CFU/mL	42.46	−0.15	−0.36
1000 CFU/mL	42.71	−0.40	−0.95
10,000 CFU/mL	42.24	0.07	0.17

**Table 4:** Average current response and relative standard deviation (%RSD) of the MIP-based electrochemical biosensor at different concentrations of *Pseudomonas aeruginosa*.

Concentration	Average (µA)	SD	% RSD
1 CFU/mL	41.08	2.34	5.71
10 CFU/mL	45.32	2.69	5.93
100 CFU/mL	56.09	6.82	12.15
1000 CFU/mL	61.23	5.54	9.05
10,000 CFU/mL	68.03	3.46	5.08

**Table 5:** Comparison of analytical performance of the proposed sensor with previously reported methods for the detection of *P. aeruginosa*.

Method	Recognition Element	LOD (CFU/mL)	Linear Range (CFU/mL)
This work	MIP (MAM/AAM/VP)	1	1–10^4^
Lectin-based electrochemical biosensor	Lectin	10	10^1^–10^6^
Fluorescence method	Dye-Based	2	10^1^–10^6^
Electrochemical sensor (review)	Various	8–18	10^1^–10^8^

## Data Availability

The original contributions presented in this study are included in the article. Further inquiries can be directed to the corresponding author.
